# The fermented soy beverage Q-CAN® plus induces beneficial changes in the oral and intestinal microbiome

**DOI:** 10.1186/s40795-021-00408-4

**Published:** 2021-03-04

**Authors:** Evangelos Dioletis, Ricardo S. Paiva, Eleanna Kaffe, Eric R. Secor, Theresa R. Weiss, Maxine R. Fields, Xinshou Ouyang, Ather Ali

**Affiliations:** 1 Internal Medicine (Digestive Diseases), Yale University School of Medicine, One Gilbert Street, TAC Bldg, Room #S241, New Haven, CT 06519 USA; 2 Hartford Hospital and University of Connecticut, Hartford, CT USA; 3 Department of Pediatrics (General Pediatrics), Yale University School of Medicine, New Haven, USA

**Keywords:** Soy, Gut microbiome, Oral microbiome, Commensals, Obesity

## Abstract

**Background:**

Soy products are associated with many beneficial health consequences, but their effects on the human intestinal microbiome are poorly characterized.

**Objectives:**

To identify the changes in the oral and fecal microbiome in lean and obese participants due to consumption of Q-CAN®, and to assess the expected consequences of these changes based on the published literature.

**Methods:**

Prospective study of lean (10) and obese (9) participants consuming Q-CAN® twice daily for 4 weeks with 8 weeks follow-up. Microbial DNA was extracted from saliva and stool samples, amplified against the V4 region of the 16S ribosomal RNA gene and data analyzed using QIIME 1.9.1 bioinformatics. Four hundred forty-four samples were collected in total, 424 of which were productive and yielded good quality data.

**Results:**

*STOOL*. In the lean population Bifidobacteria and Blautia show a significant increase while taking Q-CAN®, and there was a trend for this in the obese population. *ORAL*. There were relatively fewer major changes in the oral microbiome with an increase in the family Veillonellaceae in the lean population while on Q-CAN®.

**Conclusion:**

Q-CAN® consumption induced a number of significant changes in the fecal and oral microbiome. Most notably an increase in the stool microbiome of Bifidobacteria and Blautia, both of which are associated with positive health benefits, and in the saliva an increase in Veillonellaceae.

**Trial registration:**

This trial was registered with Clinicaltrials.gov on January 14th 2016.

ClinicalTrials.gov Identifier: NCT02656056

**Supplementary Information:**

The online version contains supplementary material available at 10.1186/s40795-021-00408-4.

## Background

Soybeans have long been recognized as sources of high-quality protein and beneficial lipids with several health benefits [1]. Consumption of fermented soybean foods is associated with many health benefits including reduced risks of type 2 diabetes (T2D) and blood pressure [2–4], and improved plasma triglyceride levels [5]. However, the mechanisms through which fermented soy may exert the above effects are unknown. One possibility is that soy nutrients are altering the microbiome of the gastrointestinal system, which is subsequently having beneficial effects. There is a considerable amount of data on the effects of soy nutrients on the microbiome of animals including mice, chickens and pigs [6, 7]. There has been great interest in the role of soy on the human gastrointestinal microbiome but there has been very limited data and using techniques that can produce results biased based on pre-existing assumptions [8–11]. To overcome these limitations, we were interested in obtaining an unbiased data set of the effects of soy products on the human gastrointestinal microbiome using 16S RNA sequencing.

Q-CAN® is a fermented soybean beverage and has been used for over 30 years as a nutritional food supplement to aid in the recovery from a wide range of conditions and is also taken during health. The beneficial effects of Q-CAN® fermented soy may be attributed to the combination of isoflavones such as Genistein and Daidzein, amino acids, trace elements, minerals, bioactive peptides and branched-chain fatty acids. Among the several putative ingredients of Q-CAN®, isoflavones were shown to exert several health benefits on the host via alterations in key bacteria that are associated with a beneficial effect [12]. In line with this, consumption of fermented soy milk was previously shown to increase healthy microbiota, such as *Bifidobacteria* and *Lactobacilli,* and to decrease the pathogenic ones, such as *Clostridia,* in healthy individuals [8, 10]. The possible restoration of the gut microbiome upon fermented soy consumption is of particular significance given that altered gut microbiome has been shown by many studies to contribute to the development and progression of cardiometabolic disorders, such as atherosclerosis, obesity, and T2D [13], NAFLD [14] and cancer [15, 16]. Q-CAN® may restore the gut and oral microbiome and through this mechanism exert its beneficial effects.

Here, we hypothesize that Q-CAN® exerts beneficial effects through reduction of pathogenic bacteria and increase of beneficial ones. Given that obesity is increasing dramatically [17] we examined a lean and an obese population and assessed the consequences of these changes based on the published literature.

## Methods

### Aim

The aim of the study was to establish the changes induced in the oral and intestinal microbiome by Q-CAN®.

### Participants

This study was approved by the Human Investigation Committee of Yale University. Subjects were recruited mostly from the campus of Yale University and had no history of abdominal surgeries (excluding cholecystectomy, appendectomy, hysterectomy, hernia repair), inflammatory bowel disease (e.g., ulcerative colitis, Crohn’s disease), GI bleeding, radiation proctitis or other known poorly controlled medical conditions that could interfere with bowel function, acute or chronic diseases, allergy to soy, soy derivatives, milk protein, alcohol use disorder, anorexia nervosa, autoimmune disease, bulimia, celiac disease, chronic infections and illicit drug use. Other exclusion criteria were major changes in dietary habits in the past 6 months, use of proton pump inhibitors, antibiotics, probiotics, laxatives (chronic use), anticholinergics, or systemic corticosteroid (within 3 months of enrollment) and medicines affected by modest dietary changes (including but not limited to, warfarin, immunosuppressives), pregnancy or history of pregnancy within the past 6 months or intent to get pregnant during study period, use of tobacco (cigarettes, smokeless tobacco, cigars, pipes) within past 30 days. Before executing this study, written informed consent was acquired from all participants. Totally 19 participants participated at the beginning. Participants were advised to maintain their normal life style during the course of the study.

#### Q-CAN® composition

Q-CAN® contained 8% fermented soy powder in water with 290.12 mg of total soy isoflavones in 240 ml and 3.6% protein. The isoflavones daidzin, genistin, daidzein, and genistein were all present at greater than 25 mg in each bottle. A profile of over 300 herbicides, pesticides and fungicides was negative and no chemical residual solvents tested were above the limit of quantification. Lead, mercury, arsenic, and cadmium were all below the detectable limit (5 to 10 parts per billion). 24-month serial assessment (accelerated and shelf) of Q-CAN® determined that soy isoflavones did not decrease in potency and microbiology analysis revealed no contamination.

### Study design

**Fig. 1:**
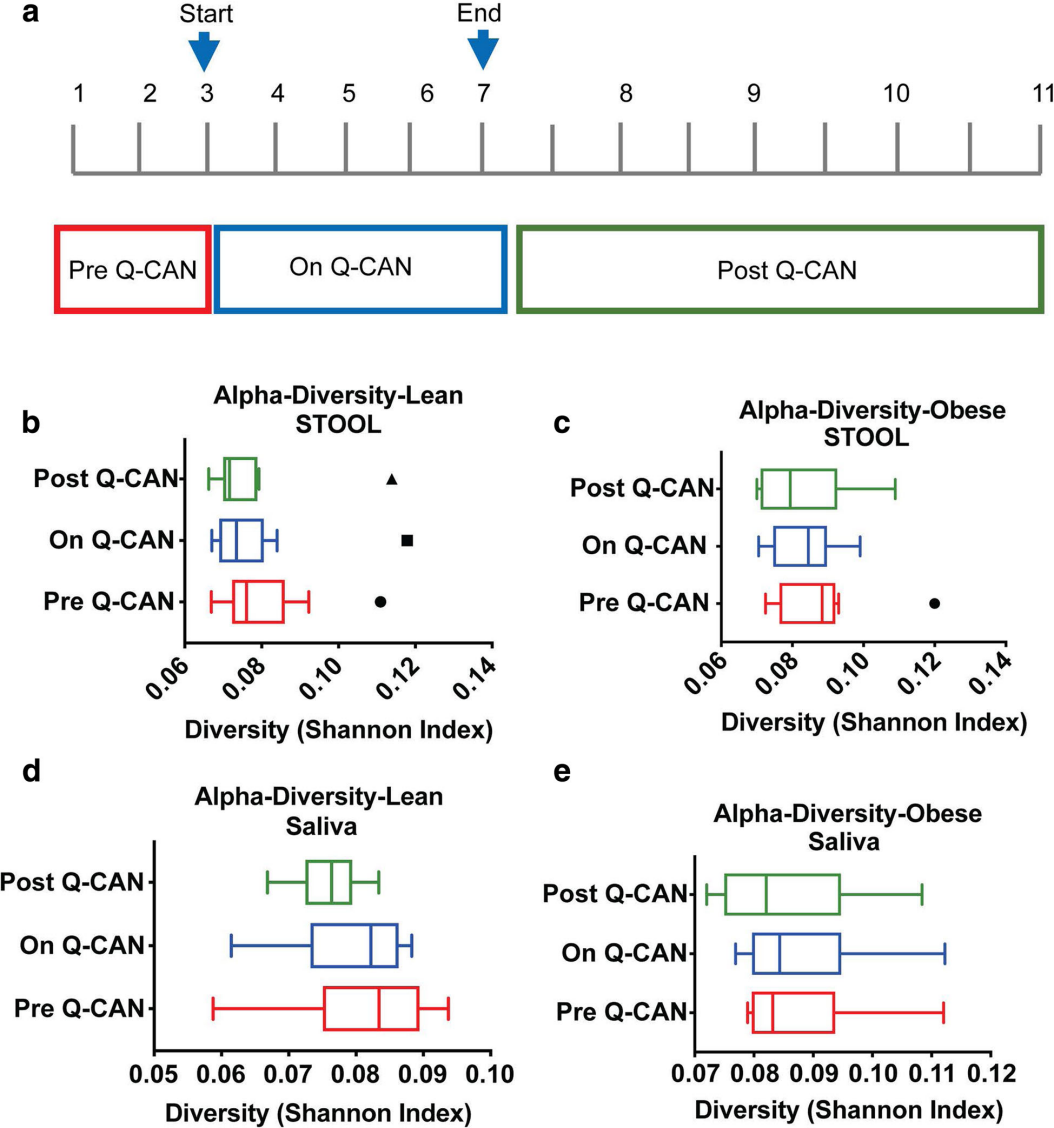
**a** Depiction of the time-frame of Q-CAN® consumption or withdrawal. **b-d** Shannon Diversity Index of intestinal or oral microbiome is not altered upon Q-CAN® consumption or withdrawal in lean or obese people. The results are the average of 3 visits in pre Q-CAN® group, 4 visits in on Q-CAN® group and 4 visits in post Q-CAN® group for each participant. Obese (*n* = 9 participants), Lean (*n* = 10 participants). The data are presented as Tukey box plots showing the median values

Twenty participants including 10 lean (3 males, 7 females, mean age 32 years, mean BMI 22 Kg/m^2^ ) and 10 obese individuals (7 males, 3 females, mean age 45 years, mean BMI 34 Kg/m^2^) were enrolled in this study. The participants performed in total 11 visits (Fig. 1a). The first 3 visits take place with one-week intervals and participants provided stool and saliva without any intervention. After the 3rd visit they started the Q-CAN® consumption (237 ml) twice daily for 4 weeks until visit 7. Every week stool and saliva were collected. At the 7th visit they stopped the Q-CAN® consumption and were monitored for 8 weeks post Q-CAN®. They gave stool and saliva samples every 2 weeks in the post Q-CAN® period. The 8-week follow up was to identify how sustained the changes induced by Q-CAN® were after consumption ceased. Three samples (2 saliva and 1 stool) were collected per visit. Saliva sample 1 was collected first thing in the morning when the participants awoke, and before brushing their teeth, eating or drinking. The stool sample was collected during the day and the saliva sample 2 was collected right after the collection of the stool sample. Saliva sample 1 was used for generating microbiome data.

### Sample collection and microbiome analysis

The saliva and stool samples were kept frozen at -80 °C until their DNA isolation. For saliva samples collection the OMNI gene-oral 501 tube was used and for stool the OMNI gene GUT OMR-200 tube. About 1 g of stool was collected from each subject in each of the 11 visits. DNA was extracted from all the samples (both saliva and stool samples from all 11 visits) according to the manufacturer instructions of the OM-501 and OMR-200 kits (DNA Genotek). All purified DNA samples were quantified via nanodrop and/or Qubit measurements. Acceptable Qubit value was > 20 ng/μl. Extracted microbial DNA from saliva and stool samples was amplified against the V4 region of the 16S ribosomal RNA gene. Raw DNA sequencing data was analyzed with the QIIME 1.9.1 bioinformatics pipeline. Samples producing > 5000 reads were considered for analysis, and the cutoff abundance was 0.01%. Statistical validation was performed using the SAS software package to calculate Least Squares Means and group difference of LSM. Four hundred forty samples were collected in total, 424 of which were productive and yielded good quality data.

The Shannon Diversity Index was calculated based on the following formula:

$$ {\displaystyle \begin{array}{c}\mathrm{s}\\ {}\mathrm{H}=\sum -\left(\mathrm{Pi}\ast \ln\ \mathrm{Pi}\right)\\ {}\mathrm{i}=1\end{array}} $$

where:

H = the Shannon diversity index.

Pi = fraction of the entire population made up of species i.

S = numbers of species encountered.

∑ = sum from species 1 to species S.

To calculate the index, the number of individuals of species found in our samples were divided by the total number of individuals of all species. This is the Pi. Afterwards, the Pi was multiplied with its natural log (P1 * ln P1). At the end, the sum of all the - (Pi * ln Pi) products is the value H (Shannon Diversity Index).

### Statistical analysis

All data are presented as the mean ± SEM. *P* < 0.05 was the level of significance. Repeated measure analyses was done on outcomes at each level, taking into account the correlation on observations occurred among the same patient. Treatment stage, gender and age were entered as fixed effects. Unstructured covariance was used. Analyses was done on each BMI level. The relative abundance of bacteria that were present at 50% of participants was also presented at the level of phylum, family, genus and species as a heat maps with hierarchical clustering using Qlucore.

## Results

**Fig. 2:**
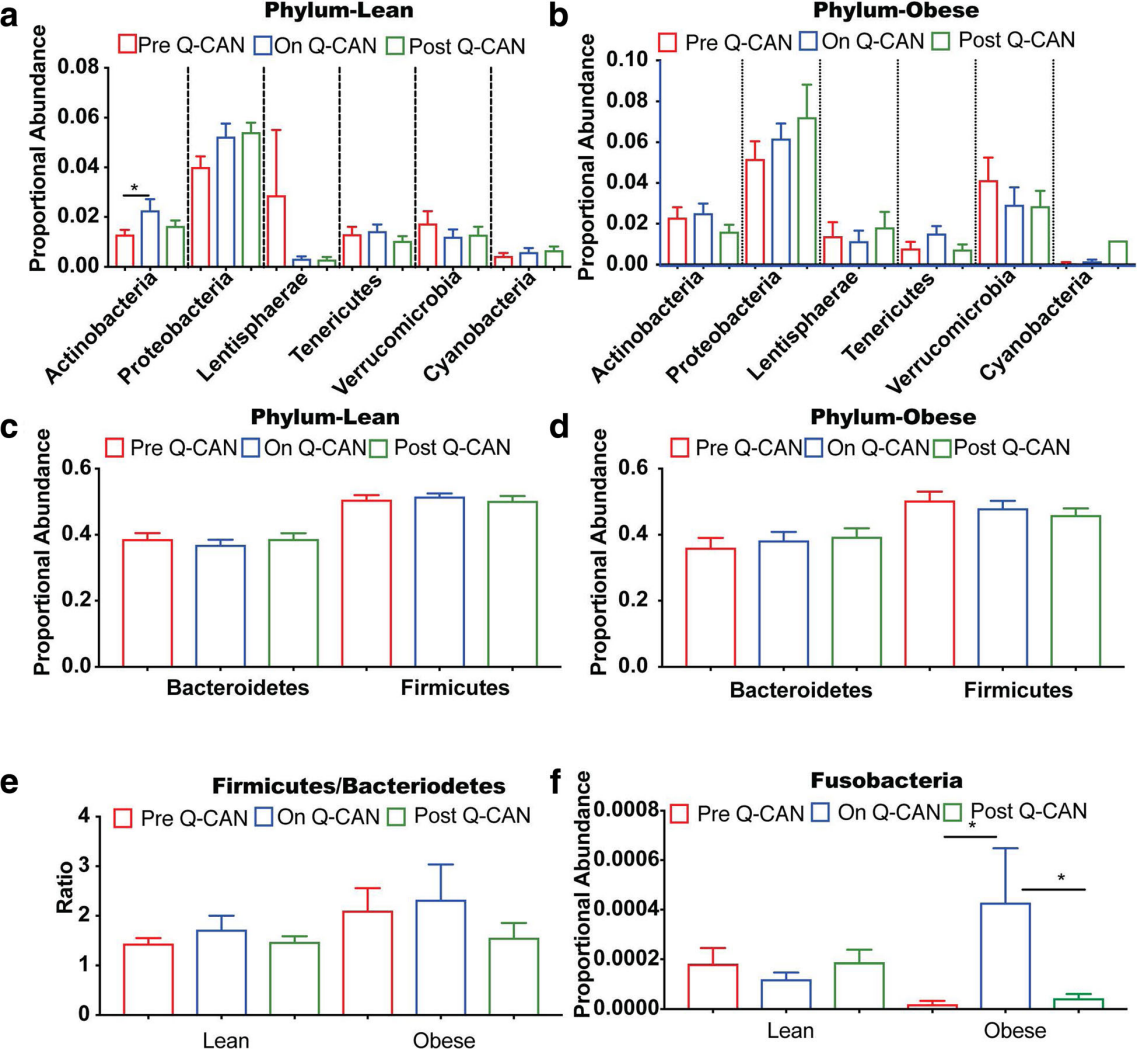
Intestinal microbiome analysis at the level of Phylum. **a-f** Bacteria at Phylum level in both lean and obese shows that only *Actinobacteria* and *Fusobacteria* are altered upon Q-CAN® consumption. **e** The ratio of *Firmicutes/Bacteroidetes* has a trend for increase in obese compared to lean ones. The results are the average of 3 visits in pre Q-CAN® group, 4 visits in on Q-CAN® group and 4 visits in post Q-CAN® group for each participant. Obese (*n* = 9 participants), Lean (*n* = 10 participants). The data are presented as median with SEM, *p < 0.05

Saliva and stool were collected from healthy, lean or obese individuals before Q-CAN® consumption (Pre Q-CAN® group), during Q-CAN® consumption (On Q-CAN® group) and after the cessation of Q-CAN® consumption (Post Q-CAN® group) (Fig. 1a). Q-CAN® consumption had no effect on alpha diversity of stool or saliva bacteria species in both obese and lean participants (Fig. 1b-e). Consumption of fermented soy product significantly increased stool *Actinobacteria* phylum populations in lean but not obese participants (Fig. 2a and b). The highest abundant phylum populations (*Firmicutes and Bacteriodetes)* and their ratio were not affected by Q-CAN® consumption or withdrawal (Fig. 2c-e). On the other hand, in the low abundant populations, Q-CAN® significantly increased *Fusobacteria* in obese participants (Fig. 2f) that returned to pre-QCAN levels upon its withdrawal. There were no other changes at the phylum level Supplementary (Figure S1).

**Fig. 3:**
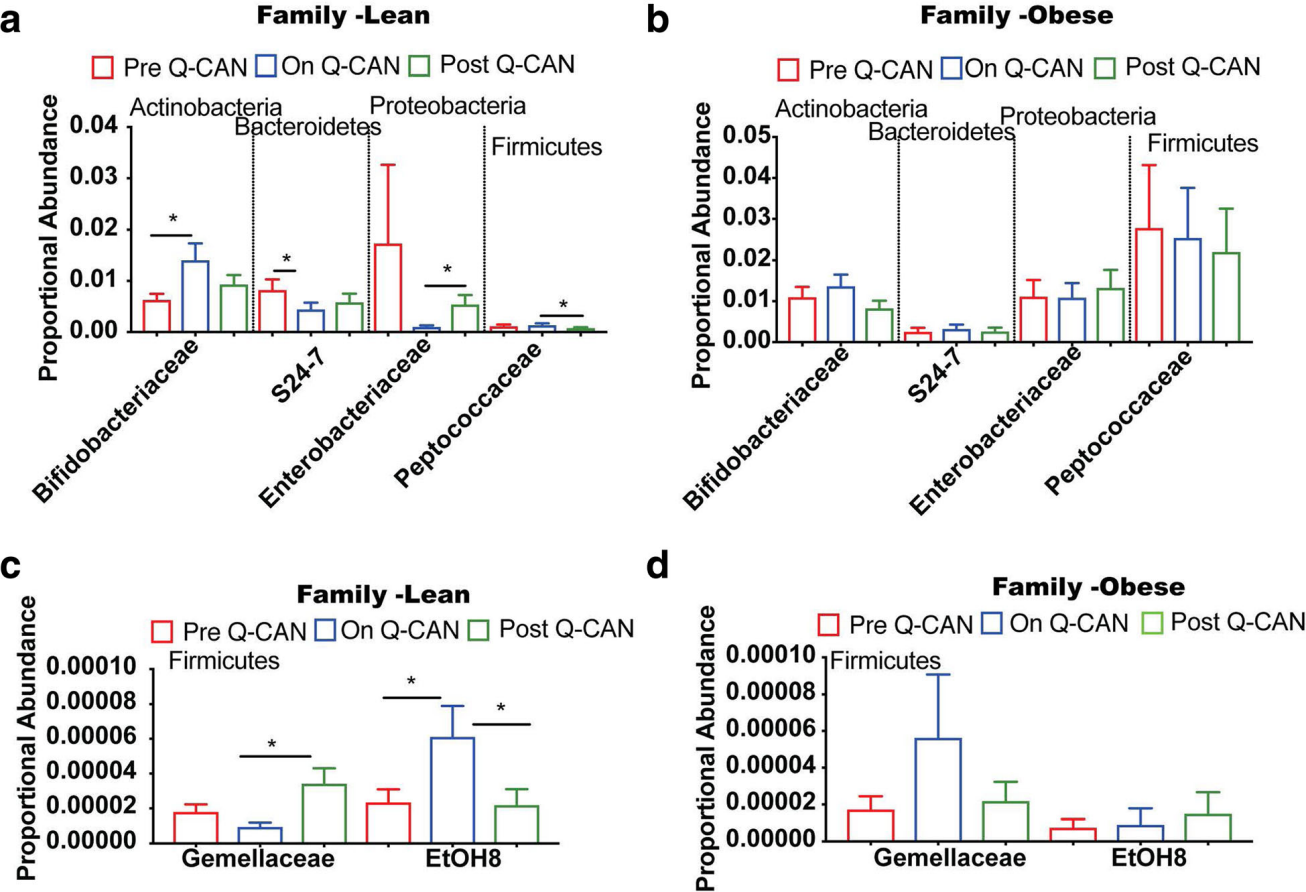
Intestinal microbiome analysis at the level of Family. A-D) Bacteria at Family level in both lean and obese shows that only *Bifidobacteriaea, S24–7* and *EtOH8* are altered upon Q-CAN® consumption. The results are the average of 3 visits in pre Q-CAN® group, 4 visits in on Q-CAN® group and 4 visits in post Q-CAN® group for each participant. Obese (*n* = 9 participants), Lean (*n* = 10 participants). The data are presented as median with SEM, *p < 0.05

At the level of Family, QCAN consumption in the lean group was associated with decreased abundance of *S24–7*, *Enterobacteriaceae* and *Gemellaceae* families (Fig. 3a, c). The decrease of the *S24–7* family was retained upon Q-CAN® withdrawal but not in *Enterobacteriaceae* and *Gemellaceae* families (Fig. 3a, c) that were significantly increased upon Q-CAN® withdrawal. Q-CAN® had opposite effect on the *Gemellaceae* family in obese group compared to lean ones (Fig. 3a, d). Several other changes were also observed in non-identified bacteria in lean and obese upon QCAN consumption or withdrawal (Supplementary Figure 2; heat map).

**Fig. 4:**
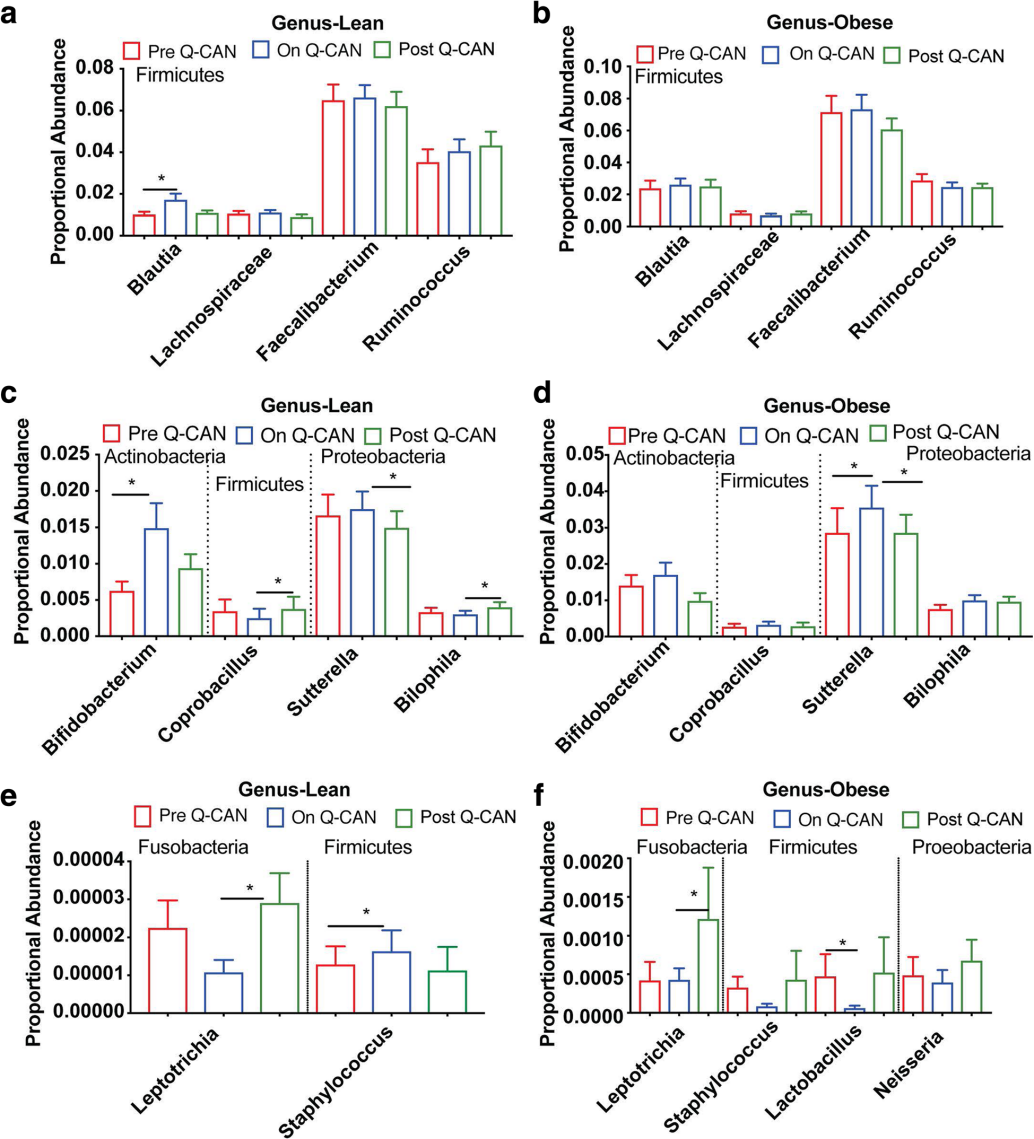
Intestinal microbiome analysis at the level of genus. **a-f** Bacteria distribution at genus level in both lean and obese participants. The levels of *Blautia, Bifidobacterium* and *Staphylococcus* genera are altered upon Q-CAN® consumption in lean and the levels of *Sutterela* and *Lactobacillus* in obese. The results are the average of 3 visits in pre Q-CAN® group, 4 visits in on Q-CAN® group and 4 visits in post Q-CAN® group for each participant. Obese (*n* = 9 participants), Lean (*n* = 10 participants). The data are presented as median with SEM, *p < 0.05

**Fig. 5:**
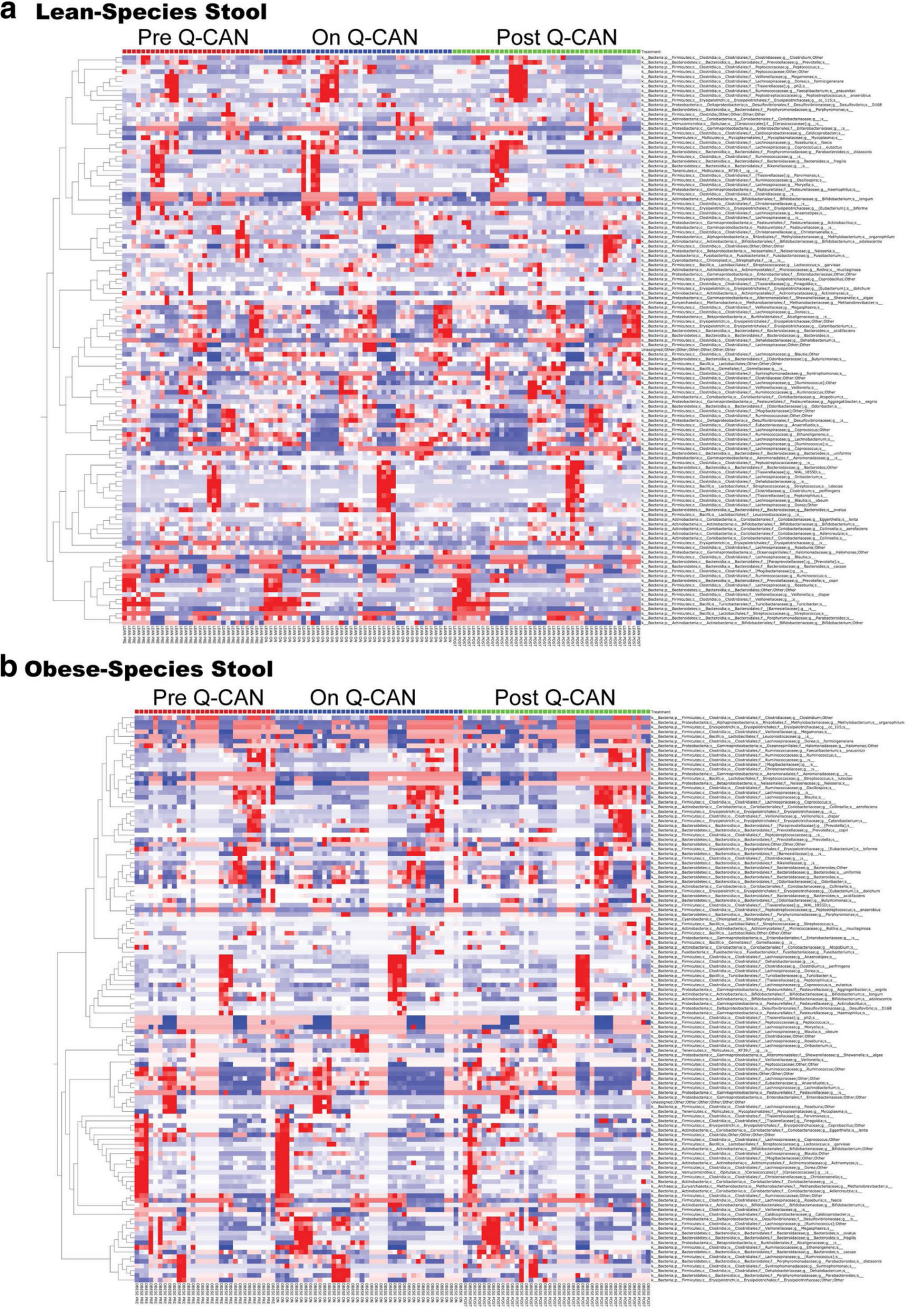
Intestinal microbiome analysis at the level of species. **a-b** Relative abundance of bacterial species is visualized by heat map. Each column represents a subject and each colored row a bacterial taxon. The intensity of the red color represents the highest abundance taxa and the intensity of the blue color the lowest abundance taxa in lean and obese people. The results are the average of 3 visits in pre Q-CAN® group, 4 visits in on Q-CAN® group and 4 visits in post Q-CAN® group for each participant. Obese (*n* = 9 participants), Lean (*n* = 10 participants)

At the genus level, the most abundant bacteria genera were not affected by Q-CAN® consumption. In the less abundant bacteria genera, Q-CAN® consumption increased the levels of *Blautia* and *Bifidobacterium* in lean participants (Fig. 4a, c). In obese, only *Sutterella* (Fig. 4d) was increased significantly upon QCAN consumption, and this was not maintained upon Q-CAN® withdrawal (Fig. 4c, d). All the species that were identified in the different visits are presented also by heat map (Fig. 5; heat map). Each column represents a subject and each row a bacterial taxon. Several other changes were also observed in non-identified genera in lean and obese upon QCAN consumption or withdrawal (Supplementary Figure 3; heat map).

**Fig. 6:**
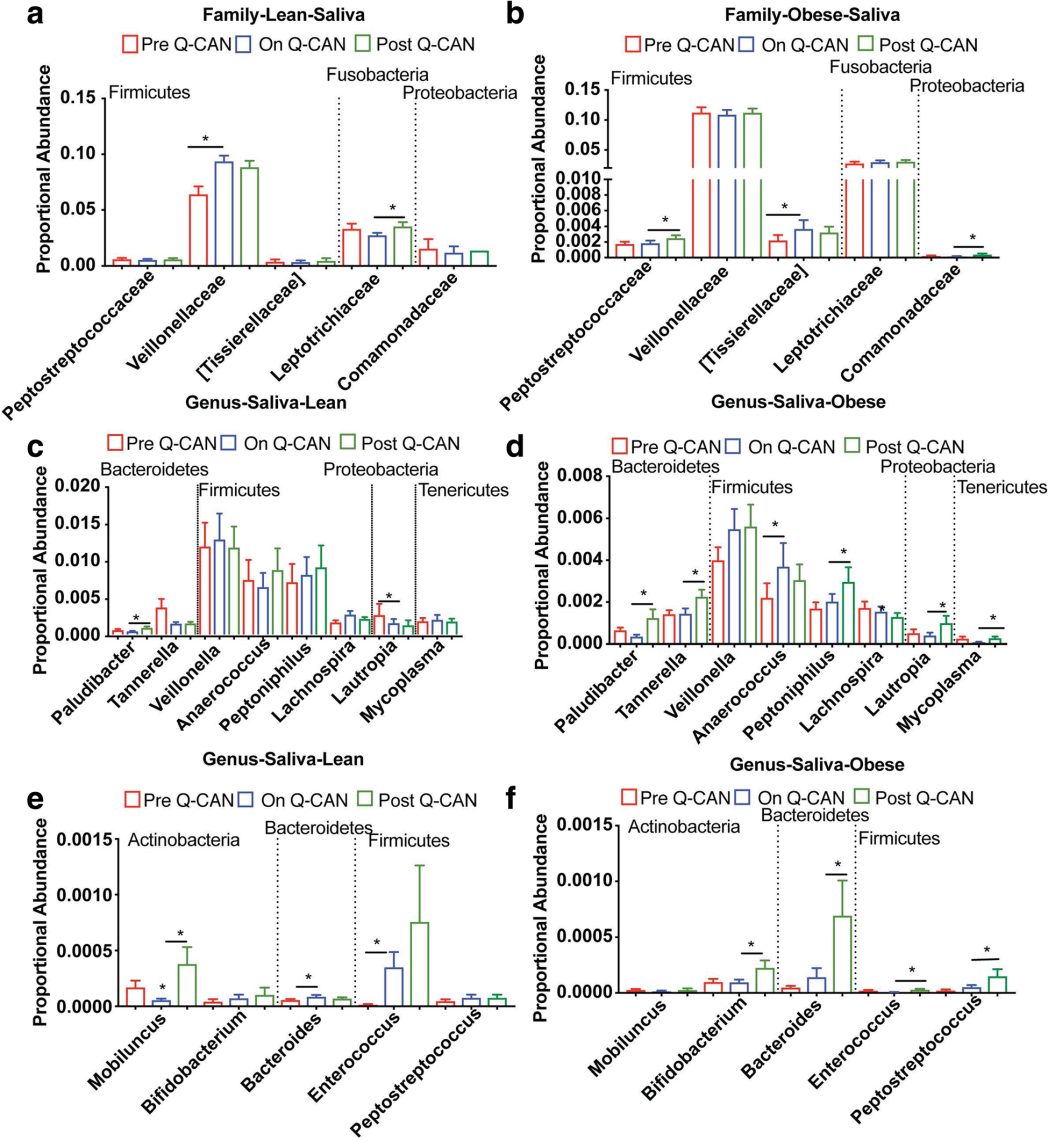
Oral microbiome analysis at the level of Family and Genus. **a**, **b** Bacteria at Family level in both lean and obese shows increase of *Veillonelleae* in lean and *Tissierallacea* in obese upon Q-CAN® consumption. **c-f** Bacteria distribution at genus level in both lean and obese participants. The results are the average of 3 visits in pre Q-CAN® group, 4 visits in on Q-CAN® group and 4 visits in post Q-CAN® group for each participant. Obese (*n* = 10 participants), Lean (*n* = 10 participants). The data are presented as median with SEM, *p < 0.05

In the saliva samples, fewer changes were observed at the phylum, family and genus level than in stool. Moreover, Q-CAN® consumption affected different oral microbes from the stool ones. At the phylum level there was no effect of Q-CAN® consumption in any of the bacteria in both lean and obese individuals (Supplementary Figure 4; heat map). At the family level, *Veillonelaceae* (Fig. 6a) was significantly increased during fermented soy consumption in the lean population. In the obese, with the exception of *Tisserelaceae* that was significantly increased during fermented soy consumption, the *Peptrosreptococcaceae* and the *Commanonadaceae* families were not affected during Q-CAN® consumption but were increased in the post Q-CAN® when compared to the on Q-CAN® group (Fig. 6b). All the bacteria at family level that were identified in the different visits are presented also by heat map (Supplementary Figure 5; heat map).

At the genus level, several genera were altered upon Q-CAN® consumption in the lean whereas in the obese the most alterations were observed upon Q-CAN® withdrawal. In the lean *Lachnospira*, *Bacteroides* and *Enterococcus* were increased upon Q-CAN® consumption whereas *Lautropia and Mobiluncus* were decreased (Fig. 6c, e). *Plaudibacter* was increased only in post Q-CAN® when compared to the on Q-CAN® group in the same population (Fig. 6c). In the obese with the exception of *Anaerococcus* that was significantly increased during fermented soy consumption, the *Plaudibacter, Tennerella, Peptoniphillus, Lautropia, Bifidobacterium, Bacteroides, Enterococcus* and *Peptosptreptococcus* were not affected during Q-CAN® consumption but were increased in the post Q-CAN® when compared to the on Q-CAN® group (Fig. 6d, e). Several other changes were also observed in non-identified genera in lean and obese upon QCAN consumption or withdrawal (Supplementary Figure 6; heat map).

**Fig. 7:**
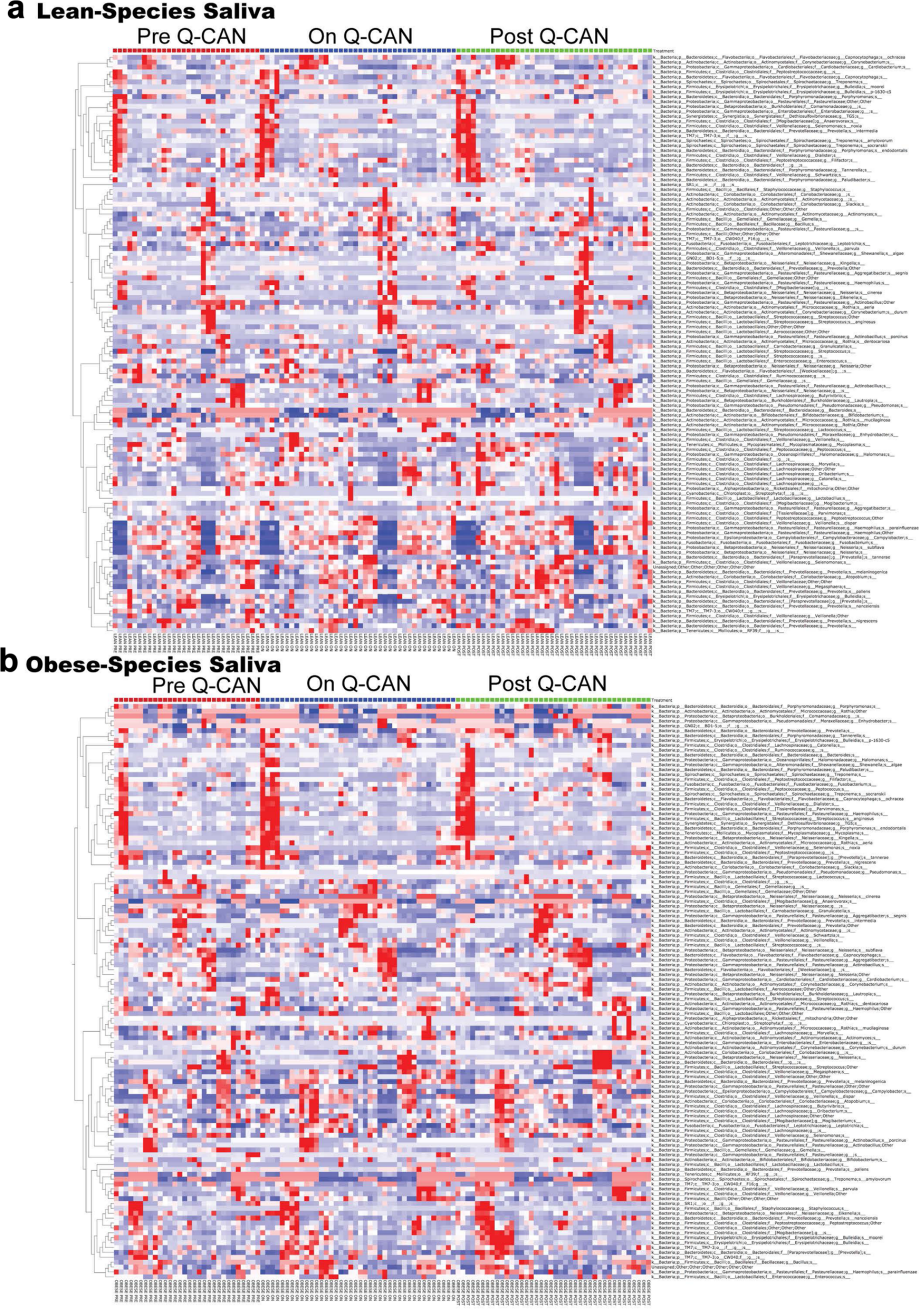
Oral microbiome analysis at the level of Species. **a-b** Relative abundance of bacterial species is visualized by heat map. Each column represents a subject and each colored row a bacterial taxon. The intensity of the red color represents the highest abundance taxa and the intensity of the blue color the lowest abundance taxa in lean and obese people. The results are the average of 3 visits in pre Q-CAN® group, 4 visits in on Q-CAN® group and 4 visits in post Q-CAN® group for each participant. Obese (*n* = 10 participants), Lean (*n* = 10 participants)

At the level of species, fewer changes were observed and they were only in non-identified bacteria (Fig. 7). All the species that were identified in the different visits are presented also by heat map (Fig. 7; heat map). Each column represents a subject and each row a bacterial taxon.

All the above changes are summarized in Table 1 where we can see that Q-CAN® consumption, compared to the Pre-QCAN®, group increases several bacteria in lean (*n* = 8 in stool and *n* = 1 in saliva) and obese (*n* = 3 in stool, *n* = 2 in saliva) participants whereas decreases few of them in lean (*n* = 2 in stool and *n* = 1 in saliva) and in obese (*n* = 2 in stool and *n* = 0 in saliva) participants. Several bacteria are also altered upon QCAN withdrawal as compared upon QCAN consumption. In the lean 5 bacteria are increased in stool and 5 in saliva whereas 3 bacteria are decreased in stool and 0 in saliva. In the obese 1 bacterium is increased in stool and 13 in saliva whereas 2 bacteria are decreased in stool and 0 in saliva.

**Table 1:** Changes in Stool and Saliva Microbiome associated with Consumption of Q-CAN®

	LEAN On vs Pre	LEAN Post vs On	OBESE On vs Pre	OBESE Post vs On
STOOL				
Phylum Level				
Actinobacteria	Increase*			
Fusobacteria			Increase*	Decrease*
Family Level				
Firmicutes;c__Clostridia;o__Clostridiales;f__Peptococcaceae		Decrease*		
Firmicutes;c__Bacilli;o__Gemellales;f__Gemellaceae	Decrease	Increase*		
Firmicutes;c__Clostridia;o__Clostridiales;f__EtOH8	Increase*	Decrease*		
Bacteroidetes;c__Bacteroidia;o__Bacteroidales;f__S24-7	Decrease*			
Actinobacteria;c__Actinobacteria;o__Bifidobacteriales;f__Bifidobacteriaceae	Increase*			
Proteobacteria;c__Gammaproteobacteria;o__Enterobacteriales;f__Enterobacteriaceae	Decrease	Incerase *		
Genus Level				
Firmicutes;c__Clostridia;o__Clostridiales;f__Lachnospiraceae;g__Blautia	Increase*	Decrease		
Firmicutes;c__Erysipelotrichi;o__Erysipelotrichales;f__Erysipelotrichaceae;g__Coprobacillus		Increase*		
Firmicutes;c__Bacilli;o__Lactobacillales;f__Lactobacillaceae;g__Lactobacillus			Decrease*	Increase
Actinobacteria;c__Actinobacteria;o__Bifidobacteriales;f__Bifidobacteriaceae;g__Bifidobacterium	Increase*	Decrease		
Proteobacteria;c__Betaproteobacteria;o__Burkholderiales;f__Alcalige0ceae;g__Sutterella		Decrease*	Increase*	Decrease*
Proteobacteria;c__Deltaproteobacteria;o__Desulfovibrio0les;f__Desulfovibrio0ceae;g__Bilophila		Increase*		
Fusobacteria;c__Fusobacteriia;o__Fusobacteriales;f__Leptotrichiaceae;g__Leptotrichia	Decrease	Increase*		Increase*
Species Level				
Bifidobacterium species	Increase*	Decrease		
Blautia species	Increase*	Decrease		
Acidaminococcus species	Increase*	Decrease		
Gemellaceae species	Decrease*	Increase*		
Parabacteroidetes species			Decrease*	Increase *
Comamonadaceae species			Increase *	Decrease
SALIVA				
Family Level				
Firmicutes;c__Clostridia;o__Clostridiales;f__Veillonellaceae	Increase*			
Firmicutes;c__Clostridia;o__Clostridiales;f__Peptostreptococcaceae				Increase *
Firmicutes;c__Clostridia;o__Clostridiales;f__[Tissierellaceae]			Increase *	
Firmicutes;c__Clostridia;o__Clostridiales;f__Ruminococcaceae				Increase *
Proteobacteria;c__Alphaproteobacteria;o__Rhizobiales;f__Methylobacteriaceae		Increase *		
Proteobacteria;c__Betaproteobacteria;o__Burkholderiales;f__Comamonadaceae				Increase *
Fusobacteria;c__Fusobacteriia;o__Fusobacteriales;f__Leptotrichiaceae		Increase *		
Genus Level				
Firmicutes;c__Bacilli;o__Lactobacillales;f__Enterococcaceae;g__Enterococcus				Increase *
Firmicutes;c__Clostridia;o__Clostridiales;f__[Tissierellaceae];g__Anaerococcus			Increase *	
Firmicutes;c__Clostridia;o__Clostridiales;f__[Tissierellaceae];g__Peptoniphilus				Increase *
Firmicutes;c__Clostridia;o__Clostridiales;f__Peptostreptococcaceae;g__Peptostreptococcus				Increase *
Firmicutes;c__Clostridia;o__Clostridiales;f__Lachnospiraceae;g__Lachnospira	Increase*			
Bacteroidetes;c__Bacteroidia;o__Bacteroidales;f__Bacteroidaceae;g__Bacteroides	Increase*			Increase *
Bacteroidetes;c__Bacteroidia;o__Bacteroidales;f__Porphyromonadaceae;g__Paludibacter		Incerase *		
Bacteroidetes;c__Bacteroidia;o__Bacteroidales;f__Bacteroidaceae;g__Bacteroides				Increase *
Bacteroidetes;c__Bacteroidia;o__Bacteroidales;f__Porphyromonadaceae;g__Paludibacter				Increase *
Bacteroidetes;c__Bacteroidia;o__Bacteroidales;f__Porphyromonadaceae;g__Tannerella				Increase *
Actinobacteria;c__Actinobacteria;o__Bifidobacteriales;f__Bifidobacteriaceae;g__Bifidobacterium				Increase *
Actinobacteria;c__Actinobacteria;o__Actinomycetales;f__Actinomycetaceae;g__Mobiluncus	Decrease	Increase*		
Proteobacteria;c__Betaproteobacteria;o__Neisseriales;f__Neisseriaceae;g__Eikenella	Decrease*	Increase *		
Proteobacteria;c__Betaproteobacteria;o__Burkholderiales;f__Burkholderiaceae;g__Lautropia				Increase *
Proteobacteria;c__Gammaproteobacteria;o__Pseudomonadales;f__Pseudomonadaceae;g__Pseudomonas				Increase *
Tenericutes;c__Mollicutes;o__Acholeplasmatales;f__Acholeplasmataceae;g__Acholeplasma		Increase *		

* denotes statistical significant

## Discussion

Fermented soy consumption has been shown to have a number of health benefits, however, the mechanisms through which fermented soy products exert these effects are totally unknown. Here we found that fermented soy beverage Q-CAN® alters the microbiome in lean, and healthy obese individuals, with a number of the changes occurring in a direction expected to improve overall health. In detail, we found that there was no effect on alpha diversity in the microbiome in the stool or saliva. There was alpha diversity however: 1) While taking Q-CAN®, at phylum level, in the stool of lean individuals there was an increase in Actinobacteria, and in obese individuals there was an increase in Fusobacteria (Fig. 2), 2) While taking Q-CAN®, at the family level in the stool of lean individuals, there was an increase in Bifidobacteriacea and EtOH8, and a decrease in S24–7 (Fig. 3), and 3) While taking Q-CAN®, at a genus level in the stool of lean individuals, there was an increase in Blautia, Bifidobacterium and Staphylococcus (Fig. 4).

There is now a large amount of data associating changes in the microbiome with physiological changes with impact on health, but very few studies in humans regarding the effects of soy products [18]. Microbiome diversity is considered to be positive and it was reassuring to see that Q-CAN® did not decrease microbiome diversity in the stool or saliva (Fig. 1b-e). The increase in Actinobacteria in lean individuals taking Q-CAN® (Fig. 2a), is of interest as Actinobacteria are one of the four major phyla of gut microbiota and has a crucial role in maintaining gut homeostasis. Actinobacteria are non-motile, multiple branching rods, gram positive, anaerobic bacteria (families: Bifidobacteria, Propionibac-teria and Corynebacteria) [19]. Within this phylum the increase was found to be in the family Bifidobacteria (Fig. 3a). Bifidobacteria have high production of short chain fatty acids (SCFA), and one of the beneficial effects of this is in the maintenance of gut barrier due to the production of butyrate [20]. Bifidobacteria can protect the host from enteropathogenic infections, such as entero-haemorrhagic *Eschericl1ia coli* and *Shigella,* and this thought to be due their high production of acetate and the biotransformation of nutrients in the diet [21–23]. This occurs via the fermentation of large polysaccharides, oligosaccarides, unabsorbed sugars and fibers. This results in the release hydrogen, carbon dioxide and SCFAs. It furthermore results in the degradation of proteins, the regulation of lipid metabolism, and the absorption and biosynthesis of vitamin K, iron, calcium and magnesium [24, 25]. Bifidobacteria are also important in the maintenance of a tolerogenic immune environment, and this is thought to be through the stimulation of intrahepatic lymphocytes [26, 27]. This is supported by an increase in gut permeability that leads to the translocation of LPS into the serum when there is a decrease in the number of Bifidobacteria [28]. This provides immune stimulation, and sustains chronic inflammatory conditions, such as insulin resistance, diabetes and liver diseases [29]. In a high fat diet mouse model, administration of *Bifidobacterium pseudocatenulatum* results in down-regulation of inflammation by reducing the production of inflammatory cytokines and chemokines, especially IL-6 and MCP-1 [30]. Overall Bifidobacteria are seen as improving gut barrier function and reduce the translocation of pro-inflammatory molecules such as lipopolysaccharide into the blood stream [28]. Bifidobacteria and Lactobacilli, are the cornerstone of many probiotic therapeutic approaches. For example a mixture of lyophilized four Lactobacilli and three Bifidobacteria strains has been demonstrated to be effective in several conditions including pouchitis, non-alcoholic steatohepatitis and in the prevention of antibiotic associated diarrhea [31–33]. Bifidobacteria treatment has also been demonstrated to improve symptoms of irritable bowel syndrome [34].

A significant increase in the phylum Actinobacteria and the family Bifidobacteria in the stool was not seen in obese individuals, however there was a trend in that direction (Fig. 2b and 3b). The lack of positive association may be due to the relatively small samples size of ten individuals in each group, and with larger samples sizes a significant increase may be seen. There was a statistically significant increase in the phylum Fusobacteria in the stool of obese individuals but this increase was not followed through at the family (Fusobacteriaceae) level and is of unclear significance. The species *Fusobacterium nucleatum* has been shown to be associated with colon cancer, although this association has not been universally reproduced [35, 36].

In lean individuals there was also an increase in the family of EtOH8 anaerobic bacteria, (Fig. 3c), but relatively little is known about the biological significance of this and it is difficult to speculate. The uncultured S24–7, a member of the Bacteroidetes family, was reduced in lean individuals while taking Q-CAN® (Fig. 3a). S24–7 are highly anaerobic bacteria that are localized to the gastrointestinal tracts of homeothermic animals and are increasingly being recognized as a numerically predominant member of the gut microbiota but due to the inability to culture them little is known about the nature of their interactions with the host [37].

At the genus level there was an increase in Blautia (family: Lachnospiraceae, order: Clostridiales, class: Clostridia, and phylum: Firmicutes) (Fig. 4a). Higher levels of Blautia have been associated with several positive health features including nutrient assimilation, immunological health, lower amount of visceral fat, reduced risk of graft versus host disease and [38–40], and administration of Blautia has been proposed as a treatment for cancer [41].

In the saliva of lean individuals there was an increase in family Veillonellaceae (phylum Firmicutes, with three genera Veillonella, Acidaminococcus, and Megasphaera) while on Q-CAN® (Fig. 6a). Members of the family Veillonellaceae are of particular interest for their probiotic effects but to date this has been investigated in animal husbandry with trials showing improvement in energy balance and inhibiting colonization by antibiotic resistance strains of bacteria [42, 43]. If it will be interesting to see if such beneficial effects are also found in the future in humans.

In addition to the phylogenetic analysis above it is important to consider analysis at a functional level by addressing changes in genes with a shared function. An example of this is the consideration of bacterial genes whose products are capable of metabolizing estrogens, identified as the estrobolome [44, 45]. A subgroup of estrogens undergoes a first passage in the liver with glucuronization or sulfunation allowing for excretion in bile, urine and feces. These estrogens can be uncombined by enteric bacterial β-glucuronidase and β-glucosidase, determining their resorption in blood circulation. At present the metabolic functions of a minority of bacterial genes has been identified. As this increases the data set presented here will be increasingly valuable and allow for analysis of Q-CAN® induced changes in the functional capacity of the microbiome.

When comparing the lean and obese populations it is clear that Q-CAN® consumption resulted in a greater number of changes in the lean than the obese (Phylum lean 1: obese 1, family lean 3: obese 0, genus lean 3: obese 0). This may be due to the microbiota of obese individuals having less diversity and therefore less opportunity for Q-CAN® to interact with a range of microbes [46–53].

## Conclusion

In conclusion, Q-CAN® induced a number of changes in the stool and saliva microbiome. The changes which were most notable and for which we currently have the greatest information on physiological impact are the increase in stool microbiome of lean participants of family Bifidobacteria which are known to have a wide variety of beneficial effects including producing SCFA, reducing intestinal permeability and improving immune function. The increase in Blautia is likewise proposed to have a number of beneficial effects including improved nutrient assimilation and reduced cancer risk. This study has also generated a significant amount of data on bacteria of unclear biological functions and this may be of value as more information is made available on the intestinal microbiome.

## Supplementary Information

**Additional file 1: Figure S1**. Intestinal microbiome analysis at the level of Phylum. A-B) Relative abundance of bacterial is visualized by heat map in both lean and obese. Each column represents a subject and each colored row a bacterial taxon. The intensity of the red color represents the highest abundance taxa and the intensity of the blue color the lowest abundance taxa in lean and obese people. The results are the average of 3 visits in pre Q-CAN® group, 4 visits in on Q-CAN® group and 4 visits in post Q-CAN® group for each participant. Obese (*n* = 9 participants), Lean (*n* = 10 participants).

**Additional file 2: Figure S2.** Intestinal microbiome analysis at the level of Family. A-B) Relative abundance of bacterial is visualized by heat map in both lean and obese. Each column represents a subject and each colored row a bacterial taxon. The intensity of the red color represents the highest abundance taxa and the intensity of the blue colour the lowest abundance taxa in lean and obese people. The results are the average of 3 visits in pre Q-CAN® group, 4 visits in on Q-CAN® group and 4 visits in post Q-CAN® group for each participant. Obese (*n* = 9 participants), Lean (*n* = 10 participants).

**Additional file 3: Figure S3.** Intestinal microbiome analysis at the level of Genus. A-B) Relative abundance of bacterial genera is visualized by heat map in both lean and obese. Each column represents a subject and each colored row a bacterial taxon. The intensity of the red color represents the highest abundance taxa and the intensity of the blue color the lowest abundance taxa in lean and obese people. The results are the average of 3 visits in pre Q-CAN® group, 4 visits in on Q-CAN® group and 4 visits in post Q-CAN® group for each participant. Obese (*n* = 9 participants), Lean (*n* = 10 participants).

**Additional file 4: Figure S4.** Oral microbiome analysis at the level of Phylum**.** A-B) Relative abundance of bacterial is visualized by heat map in both lean and obese. Each column represents a subject and each colored row a bacterial taxon. The intensity of the red color represents the highest abundance taxa and the intensity of the blue color the lowest abundance taxa in lean and obese people. The results are the average of 3 visits in pre Q-CAN® group, 4 visits in on Q-CAN® group and 4 visits in post Q-CAN® group for each participant. Obese (*n* = 10 participants), Lean (*n* = 10 participants).

**Additional file 5: Figure S5**. Oral microbiome analysis at the level of Family. A-B) Relative abundance of bacterial is visualized by heat map in both lean and obese. Each column represents a subject and each colored row a bacterial taxon. The intensity of the red color represents the highest abundance taxa and the intensity of the blue color the lowest abundance taxa in lean and obese people. The results are the average of 3 visits in pre Q-CAN® group, 4 visits in on Q-CAN® group and 4 visits in post Q-CAN® group for each participant. Obese (*n* = 10 participants), Lean (*n* = 10 participants).

**Additional file 6: Figure S6.** Oral microbiome analysis at the level of Genus**.** A-B) Relative abundance of bacterial genera is visualized by heat map in both lean and obese. Each column represents a subject and each colored row a bacterial taxon. The intensity of the red color represents the highest abundance taxa and the intensity of the blue color the lowest abundance taxa in lean and obese people. The results are the average of 3 visits in pre Q-CAN® group, 4 visits in on Q-CAN® group and 4 visits in post Q-CAN® group for each participant. Obese (*n* = 10 participants), Lean (*n* = 10 participants)

## Data Availability

Supporting data is available on request to Xinshou Ouyang at xinshou.ouyang@yale.edu

## Abbreviations

T2D: Type 2 diabetes
SCFA: short chain fatty acids
LPL: lipoprotein lipase
