# Supplementary material for: The fermented soy beverage Q-CAN® plus induces beneficial changes in the oral and intestinal microbiome
Source: BMC Nutr. 2021 Mar 4;7:6. doi: 10.1186/s40795-021-00408-4 (PMC7931600; doi:10.1186/s40795-021-00408-4)
Supplement: Supplementary file 1 — Additional file 1: Figure S1. Intestinal microbiome analysis at the level of Phylum. A-B) Relative abundance of bacterial is visualized by heat map in both lean and obese. Each column represents a subject and each colored row a bacterial taxon. The intensity of the red color represents the highest abundance taxa and the intensity of the blue color the lowest abundance taxa in lean and obese people. The results are the average of 3 visits in pre Q-CAN® group, 4 visits in on Q-CAN® group and 4 visits in post Q-CAN® group for each participant. Obese (n = 9 participants), Lean (n = 10 participants). [file 40795_2021_408_MOESM1_ESM.pdf]

FIGURE S1

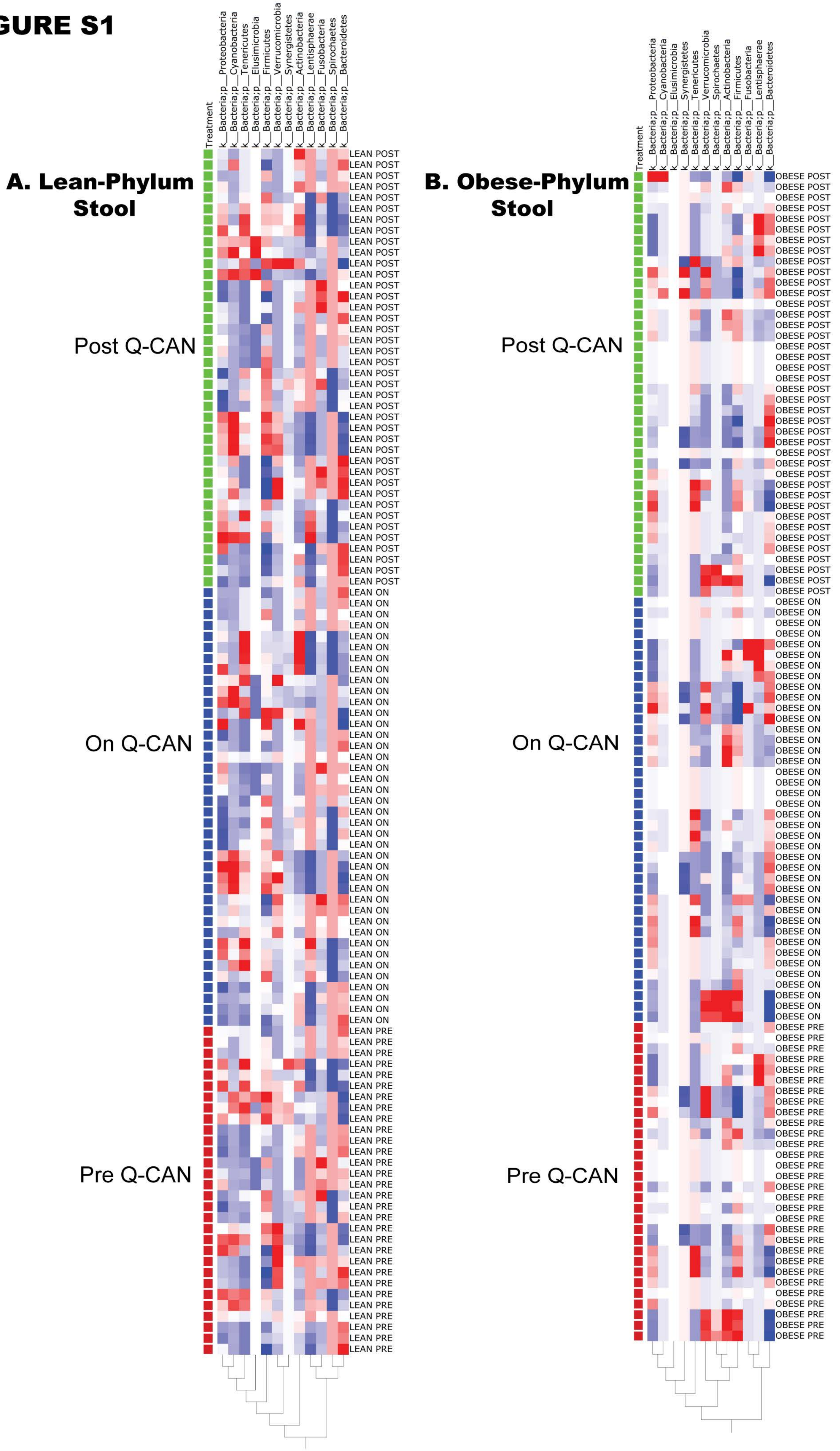
A. Lean-Phylum Stool
B. Obese-Phylum Stool
Post Q-CAN
On Q-CAN
Pre Q-CAN
Treatment
k__Bacteria;p__Proteobacteria
k__Bacteria;p__Cyanobacteria
k__Bacteria;p__Tenericutes
k__Bacteria;p__Elusimicrobia
k__Bacteria;p__Firmicutes
k__Bacteria;p__Verrucomicrobia
k__Bacteria;p__Synergistetes
k__Bacteria;p__Actinobacteria
k__Bacteria;p__Lentisphaerae
k__Bacteria;p__Fusobacteria
k__Bacteria;p__Spirochaetes
k__Bacteria;p__Bacteroidetes
