# Supplementary material for: The fermented soy beverage Q-CAN® plus induces beneficial changes in the oral and intestinal microbiome
Source: BMC Nutr. 2021 Mar 4;7:6. doi: 10.1186/s40795-021-00408-4 (PMC7931600; doi:10.1186/s40795-021-00408-4)
Supplement: Supplementary file 4 — Additional file 4: Figure S4. Oral microbiome analysis at the level of Phylum. A-B) Relative abundance of bacterial is visualized by heat map in both lean and obese. Each column represents a subject and each colored row a bacterial taxon. The intensity of the red color represents the highest abundance taxa and the intensity of the blue color the lowest abundance taxa in lean and obese people. The results are the average of 3 visits in pre Q-CAN® group, 4 visits in on Q-CAN® group and 4 visits in post Q-CAN® group for each participant. Obese (n = 10 participants), Lean (n = 10 participants). [file 40795_2021_408_MOESM4_ESM.pdf]

FIGURE S4

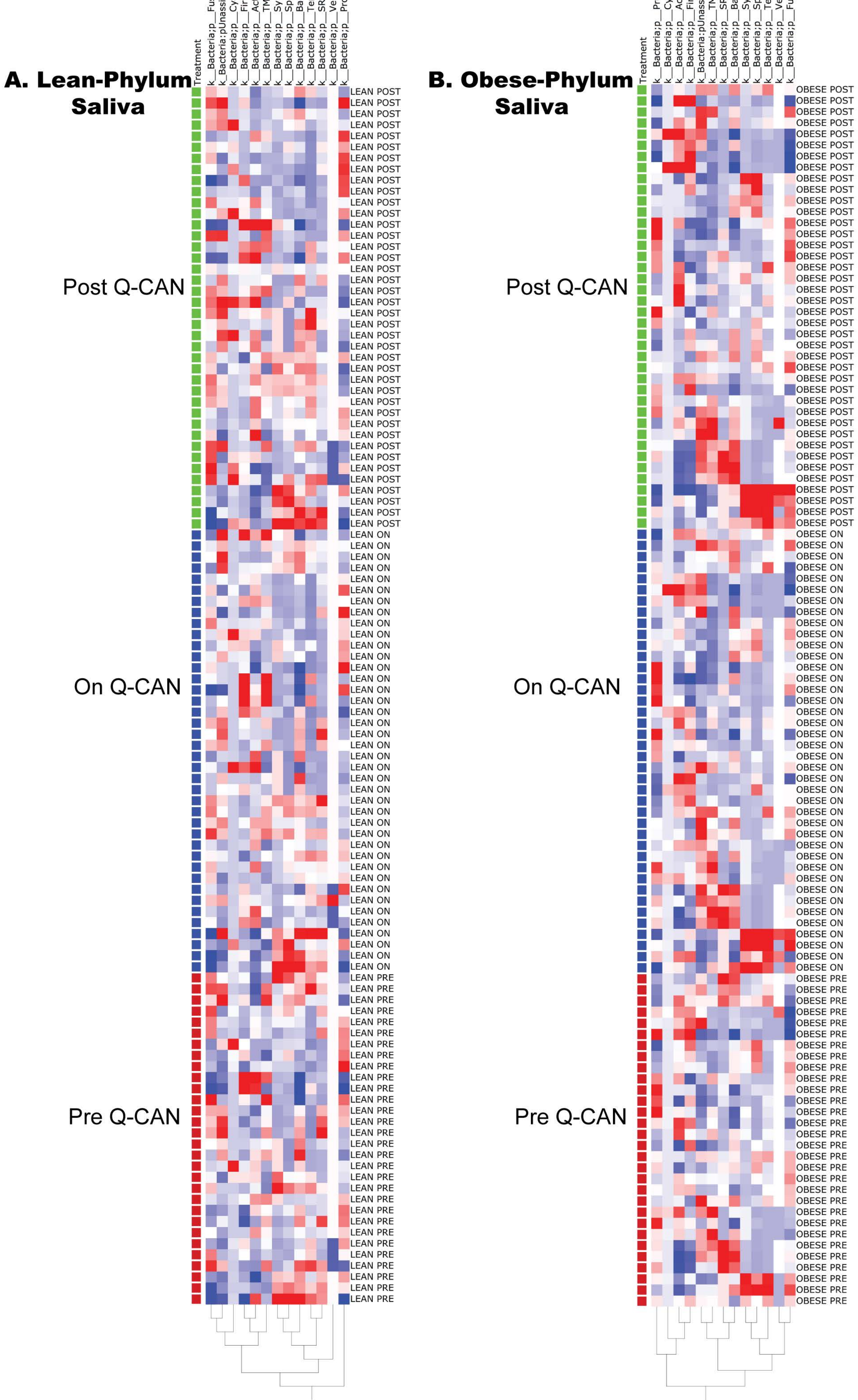
A. Lean-Phylum Saliva
B. Obese-Phylum Saliva
Treatment
k__Bacteria;p__Fusobacteria
k__Bacteria;pUnassigned;Other
k__Bacteria;p__Cyanobacteria
k__Bacteria;p__Firmicutes
k__Bacteria;p__Actinobacteria
k__Bacteria;p__TM7
k__Bacteria;p__Synergistetes
k__Bacteria;p__Spirochaetes
k__Bacteria;p__Bacteroidetes
k__Bacteria;p__Tenericutes
k__Bacteria;p__SR1
k__Bacteria;p__Verrucomicrobia
k__Bacteria;p__Proteobacteria
Post Q-CAN
On Q-CAN
Pre Q-CAN
LEAN POST
LEAN ON
LEAN PRE
OBESE POST
OBESE ON
OBESE PRE
