# Supplementary material for: The fermented soy beverage Q-CAN® plus induces beneficial changes in the oral and intestinal microbiome
Source: BMC Nutr. 2021 Mar 4;7:6. doi: 10.1186/s40795-021-00408-4 (PMC7931600; doi:10.1186/s40795-021-00408-4)

FIGURE S6

A Lean-Genus Saliva

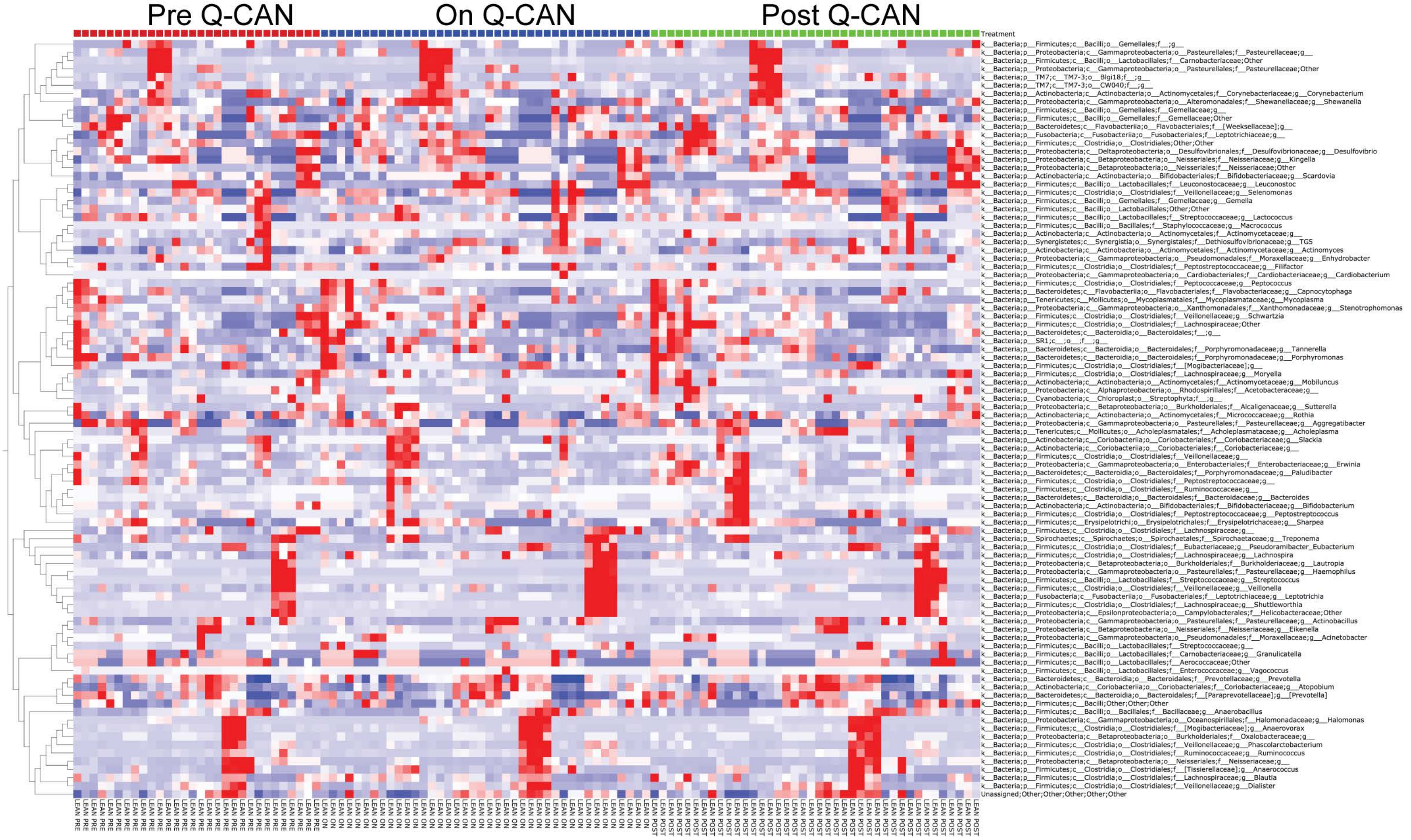

B Obese-Genus Saliva

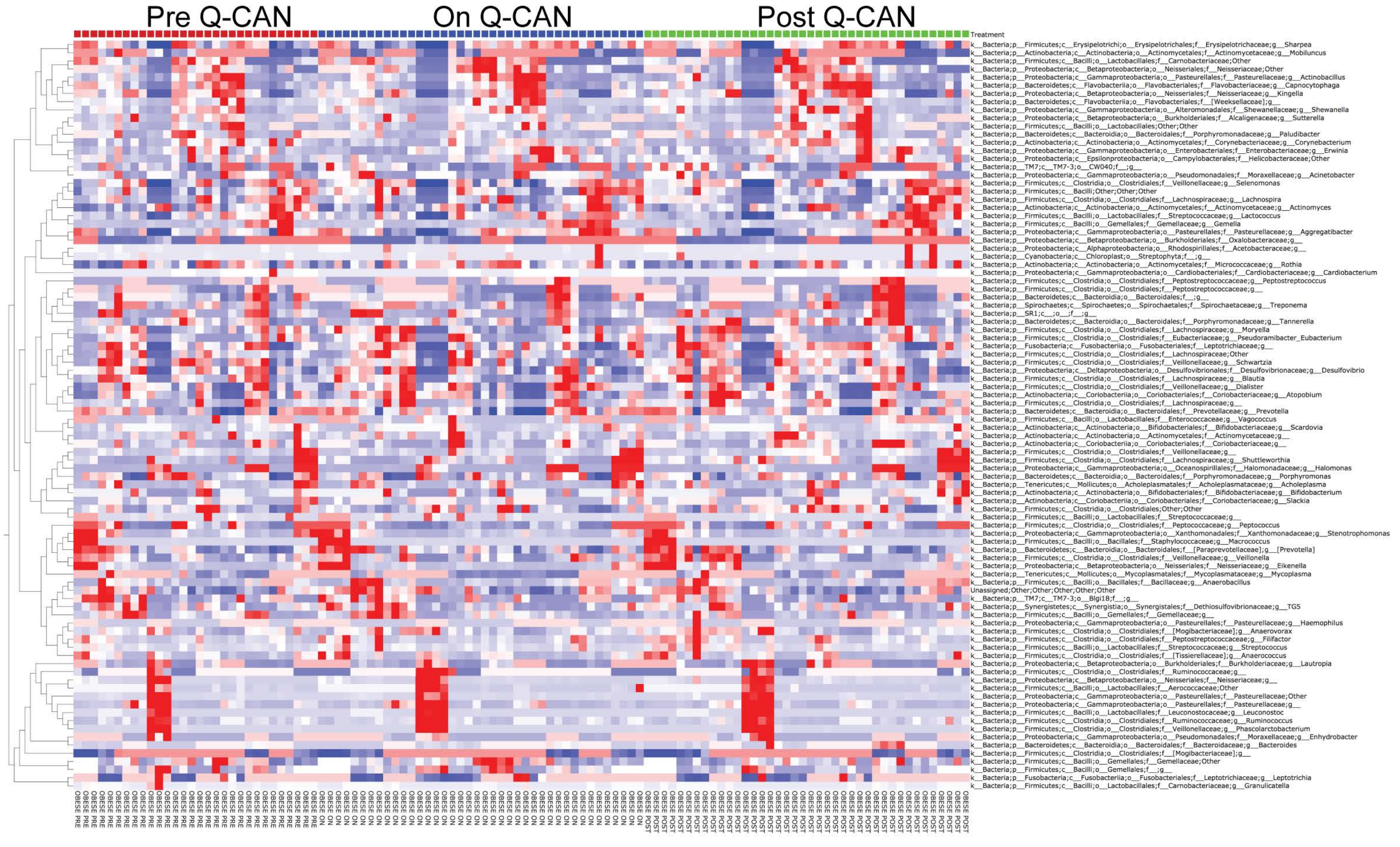

Supplement: Supplementary file 6 — Additional file 6: Figure S6. Oral microbiome analysis at the level of Genus. A-B) Relative abundance of bacterial genera is visualized by heat map in both lean and obese. Each column represents a subject and each colored row a bacterial taxon. The intensity of the red color represents the highest abundance taxa and the intensity of the blue color the lowest abundance taxa in lean and obese people. The results are the average of 3 visits in pre Q-CAN® group, 4 visits in on Q-CAN® group and 4 visits in post Q-CAN® group for each participant. Obese (n = 10 participants), Lean (n = 10 participants) [file 40795_2021_408_MOESM6_ESM.pdf]
